# Codevelopment of a Text Messaging Intervention to Support Adherence to Adjuvant Endocrine Therapy in Women With Breast Cancer: Mixed Methods Approach

**DOI:** 10.2196/38073

**Published:** 2023-05-24

**Authors:** Sophie M C Green, David P French, Louise H Hall, Yvonne Kiera Bartlett, Nikki Rousseau, Erin Raine, Catherine Parbutt, Benjamin Gardner, ROSETA Investigators, Samuel G Smith

**Affiliations:** 1 Leeds Institute of Health Sciences University of Leeds Leeds United Kingdom; 2 Manchester Centre for Health Psychology University of Manchester Manchester United Kingdom; 3 Leeds Institute of Clinical Trials Research School of Medicine University of Leeds Leeds United Kingdom; 4 St James's University Hospital Leeds Teaching Hospitals National Health Service Trust Leeds United Kingdom; 5 Department of Psychology Institute of Psychiatry, Psychology and Neuroscience King's College London London United Kingdom

**Keywords:** breast cancer, medication adherence, habit formation, behavior change techniques, SMS text messages, intervention development

## Abstract

**Background:**

Adjuvant endocrine therapy (AET) reduces breast cancer recurrence and mortality in women with early-stage breast cancer. Unintentional nonadherence to AET is common (eg, forgetting to take medication). Forming habits surrounding medication taking could reduce reliance on memory and improve AET adherence. SMS text messaging interventions may offer a low-cost approach for promoting medication-taking habits. To optimize the likely effectiveness of such SMS text messages, the content should be developed using a transparent approach to ensure fidelity to relevant psychological theory and with user input to increase acceptability.

**Objective:**

This study aimed to develop a pool of brief SMS text messages promoting habit formation to support AET adherence, which are acceptable to women with breast cancer and show fidelity to theory-based behavior change techniques (BCTs).

**Methods:**

According to published literature, we selected 6 BCTs derived from the habit formation model: action planning, habit formation, restructuring the physical environment, adding objects to the environment, prompts/cues, and self-monitoring of behavior. In study 1, behavior change experts (n=10) created messages, each based on 1 of the 6 BCTs, in a web-based workshop and rated the fidelity of the messages to the intended BCT. In study 2, women with experience of taking AET discussed the acceptability of the messages in a focus group (n=5), and the messages were refined following this. In study 3, women with breast cancer rated the acceptability of each message in a web-based survey (n=60). In study 4, additional behavior change experts rated the fidelity of the remaining messages to the intended BCT in a web-based survey (n=12). Finally, a consultant pharmacist reviewed a selection of messages to ensure that they did not contradict general medical advice.

**Results:**

In study 1, 189 messages were created targeting the 6 BCTs. In total, 92 messages were removed because they were repetitious, unsuitable, or >160 characters, and 3 were removed because of low fidelity (scoring <5.5/10 on a fidelity rating scale). Following study 2, we removed 13 messages considered unacceptable to our target population. In study 3, all remaining messages scored above the midpoint on an acceptability scale (1-5); therefore, no messages were removed (mean 3.9/5, SD 0.9). Following study 4, we removed 13 messages owing to low fidelity (scoring <5.5/10 on a fidelity rating scale). All the remaining messages showed fidelity to the intended BCTs (mean 7.9/10, SD 1.3). Following the pharmacist review, 2 messages were removed, and 3 were amended.

**Conclusions:**

We developed a pool of 66 brief SMS text messages targeting habit formation BCTs to support AET adherence. These showed acceptability to women with breast cancer and fidelity to the intended BCTs. The delivery of the messages will be further evaluated to assess their effect on medication adherence.

## Introduction

### Background

Adjuvant endocrine therapy (AET) is routinely prescribed for 5 to 10 years to women with early-stage estrogen receptor–positive (ER+) breast cancer to reduce recurrence and mortality [1-4]. It is an oral tablet taken once daily. Despite its benefits, up to three-fourths (75%) of women do not take AET as prescribed [5-7], with up to half of women discontinuing it within 5 years [6,8]. Nonadherence to AET increases the risk of breast cancer–related recurrence and mortality and increases health care costs [4,9,10].

Nonadherence to AET can be intentional or unintentional. Intentional reasons for nonadherence include difficulties with side effects, concerns about AET, and psychological distress [11-15]. Unintentional reasons include forgetfulness, being away from home, and difficulty refilling a prescription [16]. Unintentional nonadherence is reported more frequently than intentional nonadherence in women with breast cancer taking AET [16,17]. Subjective cognitive impairment, including impaired memory, is commonly reported by breast cancer survivors following chemotherapy [18,19], which may contribute to unintentional nonadherence [7,12,20,21].

A Cochrane review of 182 randomized controlled trials on medication adherence interventions across multiple long-term conditions highlighted the need for more effective and novel interventions to support medication adherence [22]. Existing interventions supporting AET adherence tend to focus on educating women about AET and breast cancer and often solely use written information [23-26]. Most of these interventions do not focus on unintentional barriers to adherence, despite their prevalence [26]. Most existing interventions have been minimally effective in improving adherence [23-26].

Making medication-taking behaviors habitual could address unintentional nonadherence, as habits reduce reliance on memory. A habitual behavior is an action triggered by exposure to a contextual cue [27]. Habitual behaviors are learned through a process of context-consistent repetition: consistently repeating a behavior (eg, taking medication) in the presence of a specific context cue (eg, a time of day) strengthens a specific cue-behavior association [28-33]. Habit theory suggests that although initial performances may require conscious effort, as the association is reinforced, the behavior can be activated automatically with minimal dependence on conscious memory or attention [30]. The formation of a habit is initially rapid, plateauing over time as the habit is formed [33,34]. Although there is individual variation in the timescale in which a behavior becomes habitual, habit formation tends to occur most notably within the first 2 weeks of attempting to change the behavior [33,35,36]. This highlights the need for support in the early phases of a behavior change intervention aimed at supporting habit formation [31,34].

According to habit theory, if habits for medication-taking behaviors are formed, taking medication could become more automatic, thereby reducing reliance on memory or the motivation to take AET [37]. In a meta-analysis including 771 interventions supporting medication adherence in a variety of clinical contexts, larger effect sizes were observed for habit-based interventions than for those using simple prompts or educational materials alone [38,39]. However, a recent review highlighted that many medication adherence interventions described as “habit based” are not theoretically informed and do not promote the process of context-consistent repetition, which is fundamental to habit formation [40].

SMS text messaging interventions are a low-cost method for supporting habit formation. They can serve as an initial reminder to take the medication and could lead to sustained behavior change through prompting medication taking in the same context repeatedly to support habit formation [41]. Meta-analyses of SMS text messaging interventions in other long-term conditions have reported positive effects on adherence (odds ratios 1.39-2.11) [42,43]. However, to date, no habit-based interventions to support medication adherence have exclusively used SMS text messages [40].

Outside of habit-based interventions, SMS text messaging interventions aimed at improving adherence to AET among women with breast cancer have shown mixed results [44-47]. In these interventions, the same messages are often repeated, potentially causing response fatigue [45,46]. The use of simple prompts in some of these interventions fails to make use of the range of known behavior change strategies and, therefore, may be unlikely to produce sustained behavior change once the messages have ceased [46]. Behavior change techniques (BCTs) are described as irreducible, active ingredients of an intervention that can be used to label the content of behavioral interventions [48]. Existing SMS text messaging interventions based on simple prompts use only 1 of the 93 BCTs identified in version 1 of the behavior change taxonomy (BCTTv1) [48]. Additional BCTs, beyond prompts and cues, should be used to promote sustained behavior change.

The message development processes for existing SMS text messaging–based interventions targeting AET adherence generally lack detail and transparency, and there is rarely a theoretical justification for the message content. Intervention development guidance highlights the importance of having a theoretical basis for an intervention and advocates for detailed reporting of intervention development to improve transparency [49-52]. Therefore, we sought to address these limitations in the field by developing SMS text messages based on specific, theory-based BCTs chosen to target habit formation, in line with habit theory [34].

### Objectives

**Figure 1:**
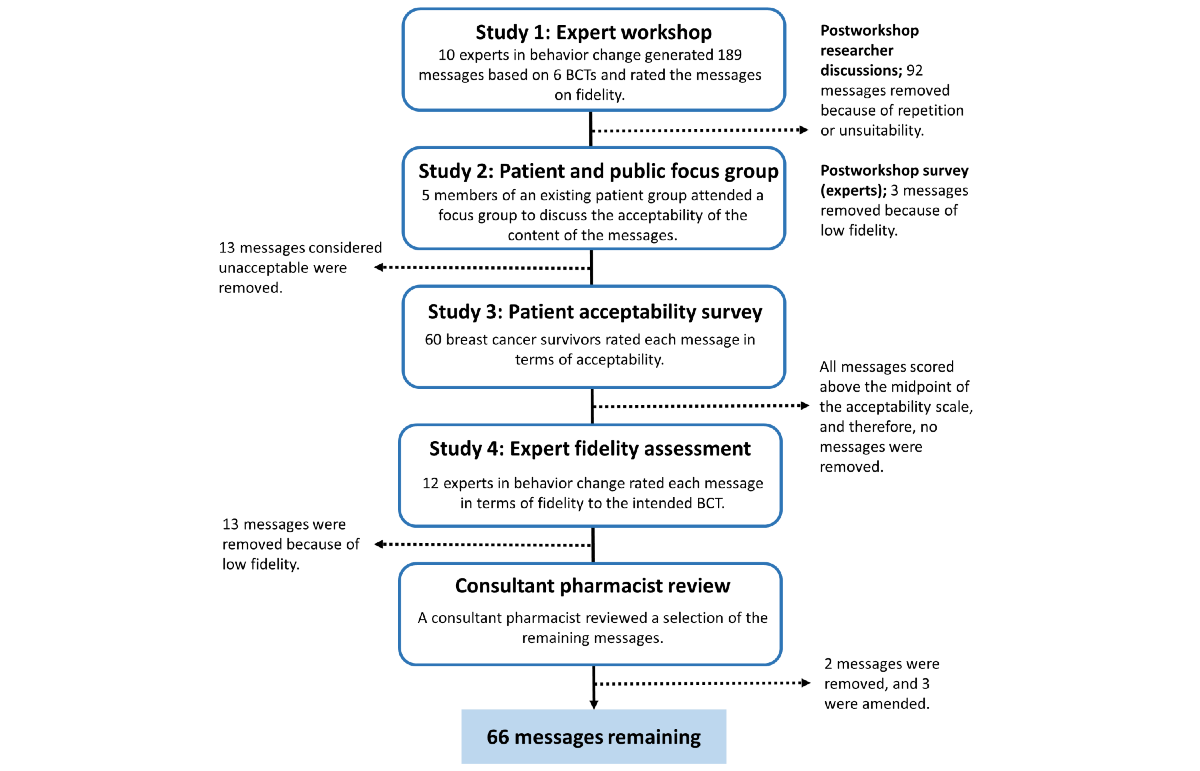
Development of SMS text messages. BCT: behavior change technique.

Using an established process [53,54], we aimed to develop a pool of brief SMS text messages that promote habitual medication taking, are acceptable to patients, and show fidelity to explicit BCTs. This process involved (1) an expert workshop to create the messages, (2) a patient focus group to determine message acceptability, (3) a web-based patient survey to assess message acceptability, and (4) a web-based expert survey to assess the fidelity of the final messages to the intended BCTs (Figure 1). The studies in this paper form part of a broader research program, which aimed to develop and optimize a multicomponent intervention to support adherence to AET in women with breast cancer [26,55]. The multicomponent intervention includes 4 components targeting a range of known barriers to AET adherence. SMS text messages targeting memory and forgetfulness are one of these components [26,55].

## Methods

In preparing this manuscript, we followed the guidance for reporting intervention development studies in health research [52].

### Target Behavior

Using the Target, Action, Context, Time framework for behavior specification [56], the target behavior is defined as women with breast cancer prescribed AET (target), taking their AET tablet (action) daily (time) in a consistent context (context). To achieve the behavior of adherence (ie, taking AET daily), ordering and collecting prescriptions is also required. Therefore, we targeted these associated behaviors to facilitate the performance of the overall behavior of taking medication daily.

### Selection of BCTs

On the basis of evidence suggesting that habit interventions could be effective for improving medication adherence [38,39] and the potential for habitual medication taking to reduce forgetfulness of taking AET, SMS text messages were designed based on habit theory. The process of habit formation involves multiple stages: deciding to undertake a behavior, initiating and repeating the behavior, and acting consistently in the same context [28-33]. Although the first 2 stages are necessary for any long-term behavior change attempt, the final stage promotes the formation of cue-behavior associations, which is unique to habit formation [29,32]. On the basis of published guidance, we selected 6 BCTs from the BCTTv1 to be used in the intervention [29,32,34,57]. Using BCTTv1 enabled us to develop a theoretically informed intervention specified in a way that will improve generalizability and replicability. The 6 chosen BCTs included a BCT explicitly promoting context-dependent repetition (*habit formation [also known as context-dependent repetition]*), BCTs promoting the use of feasible environmental cues for medication taking (*prompts/cues*, *restructuring the physical environment*, and *adding objects to the environment* [32,34,57]), a BCT specifying behavioral responses to these cues (*action planning*) [34,57,58], and a BCT promoting monitoring successful implementation of these responses (*self-monitoring of behavior* [34,48,57]). The BCTs were identified by 1 author (SMCG) and discussed and agreed by consensus with 4 other members of the research team (SGS, DPF, YKB, and ER).

### Development Studies

We followed an established process using a series of studies to develop the pool of SMS text messages [53,54]. This approach involved developing SMS text messages based on theory with experts in behavior change before gaining qualitative and quantitative feedback from the patient population. The final stage involved checking whether the messages still targeted the BCTs they intended to target after any adaptations had been made.

### Study 1: Expert Workshop

#### Aim

The aim was to generate 12 to 15 messages for each of the 6 chosen BCTs targeting habit formation and to assess message fidelity to the intended BCTs.

#### Participants

We emailed 25 UK experts in behavior change, identified through the research team’s networks and by searching university websites and Twitter profiles. We sent a reminder email to nonrespondents after 1 week. The participants were given a £100 honorarium for their time. A currency exchange rate of GBP £1=US $1.25 is applicable.

#### Procedure

We sent a web-based questionnaire asking for e-consent, demographic information, and expertise in behavior change. Participants who completed this questionnaire were sent the schedule for the 1-day web-based workshop. The schedule included the aims for the day, target behaviors for the messages, and the names and descriptions of the 6 target BCTs. The workshop was hosted on Zoom (Zoom video communications) and was split into 3 sections. For each section, participants were split into 2 groups (5 participants per group), facilitated by 2 researchers per group (SMCG, SGS, ER, and LHH). Each group was introduced to a BCT with a short description and an example taken from the BCTTv1 [48]. Participants were informed that messages must be <160 characters to fit in a single SMS text message. Participants were asked to generate SMS text messages to support AET adherence and enter the messages into a web-based real-time platform to collate uploaded content (Padlet). Participants then had 15 minutes to discuss the SMS text messages and BCT in their group. SMS text messages could be modified within this time, and new suggestions were added. This process was repeated 3 times such that across 2 groups, messages were generated for all 6 BCTs. The experts were allocated to a different group after each round of message generation. One workshop participant is an author of this manuscript (BG).

Following the workshop, 4 research team members (SMCG, SGS, ER, and LHH) removed messages deemed unsuitable for the intervention (eg, they were substantially >160 characters or made unrealistic suggestions that could not be actioned by the patient). Then, each researcher rated each message based on how well it addressed the target behaviors on a scale of 1 to 10 (coherence to the behavior).

Two working days after the workshop, the participants were sent a survey containing the remaining messages. Participants were asked to rate how relevant they felt the BCT was to support medication adherence within this population, how well they thought the aim of generating 12 to 15 good-quality messages had been achieved, and the fidelity of each message to the BCT it was intended to target. All 3 questions were assessed on a scale of 1 (not relevant or not very well) to 10 (very relevant or very well).

#### Analysis

We removed messages scoring <5.5 on the research team’s coherence rating, as this was the midpoint of the scale. Participant characteristics were summarized. We calculated the means and SDs of each SMS text message on fidelity, and an a priori threshold of <5.5 on the fidelity scale was used to remove messages, as this was the midpoint on the scale and has been used as a cutoff in a previous similar study [53]. We summarized the responses regarding the relevance of the BCT and the aim of generating 12 to 15 good-quality messages per BCT.

### Study 2: Focus Group

#### Aim

The aim was to assess the acceptability of the SMS text messages generated in study 1 to women taking AET.

#### Participants

Participants were recruited from a preexisting patient and public involvement group. Patient and public involvement group members were originally recruited via an advertisement circulated in a newsletter for a charity that supports people with cancer. All members had a diagnosis of ER+ breast cancer and were currently taking AET. All members were offered £37.50 compensation for their time, in line with the published guidelines [59].

#### Procedure

All participants completed demographic and clinical questions and were sent a copy of the SMS text messages 2 weeks before the meeting. The focus group was hosted on Microsoft Teams, lasted 90 minutes, and was facilitated by SMCG and ER. Each BCT was presented along with a short description taken from the BCTTv1 and the SMS text messages relating to that BCT. The participants were asked to discuss the acceptability of the messages and suggestions for rewording. A topic guide was used to structure the discussion (Multimedia Appendix 1).

#### Analysis

The focus group was recorded and transcribed verbatim. One author (SMCG) extracted all suggestions from the transcript and made amendments to the messages when there was no disagreement between participants. If there was disagreement among participants, these instances were discussed within the research team (SMCG, ER, SGS, and LHH). Where the research team could not reach an agreement, the message was retained and included in study 3.

### Study 3: Patient Survey

#### Aim

The aim was to determine the acceptability of SMS text messages remaining after studies 1 and 2.

#### Participants

In total, 60 women diagnosed with ER+ breast cancer were recruited via a recruitment company, Dynata, to participate in a web-based survey. Dynata provided the women with a small monetary incentive on completion of the survey.

#### Procedure

Dynata sent the web-based survey link to their panel members. The survey contained information on the study, an e-consent form, and demographic questions. Participants who were eligible and provided e-consent were asked to rate the SMS text messages based on their acceptability, with responses on a 5-point Likert scale (1=completely unacceptable and 5=very acceptable). The order in which the messages appeared was randomized.

#### Analysis

We summarized participant demographics, means, and SDs for the acceptability of each SMS text message. We calculated an acceptability score for each BCT by averaging the acceptability scores of the messages related to that BCT. On the acceptability scale, any messages scoring below an a priori threshold of 3 were removed, as this was the midpoint of the scale and has been used as a cutoff in a previous similar study [53].

### Study 4: Expert Survey

#### Aim

The aim was to assess the fidelity of the remaining SMS text messages to the intended BCTs.

#### Participants

We emailed 41 UK experts in behavior change. This included 15 experts who had not responded to the study 1 invitation or could not attend the workshop. In total, 26 additional potential participants were identified from the research team’s networks and through further searching of university websites and Twitter profiles. Study 1 participants were ineligible and were not contacted. A £25 Amazon voucher was offered upon completion of the survey.

#### Procedure

We emailed a link to a web-based survey containing information about the study and an e-consent form. If the participants consented, they were asked to rate each message on fidelity to the intended BCT on a scale of 1 (not very well) to 10 (very well). A description and an example from the BCTTv1 were provided.

#### Analysis

We summarized participant demographics and the means and SDs for each text message and BCT. We removed messages scoring below an a priori threshold of 5.5 on fidelity, as this was the midpoint of the scale and has been used as a cutoff in a previous similar study [53].

### Clinical Review

A selection of 20 messages were sent to a consultant clinical pharmacist with experience of AET to ensure advice in the messages was appropriate and not conflicting with general medical advice. The consultant pharmacist was a member of the trial management group for the larger program of research that this study is part of. The 20 messages to be reviewed included all messages in which there could be any risk of conflicting or dangerous advice. The review occurred at this stage to ensure that the final versions of the messages were checked.

### Ethics Approval, Informed Consent, and Participation

Ethics approval was granted by the University of Leeds School of Medicine Ethics Committee (MREC 20-038 July 2021). All procedures performed in studies involving human participants were in accordance with the ethical standards of the institutional research committee and with the 1964 Declaration of Helsinki and its later amendments or comparable ethical standards. Informed consent was obtained from all the participants included in this study. All data from the study were deidentified. Participants were compensated in the following ways: study 1: £100, study 2: £37.50, study 3: small incentive provided directly from the market research company, and study 4: £25 Amazon voucher.

## Results

The flowchart of the 4 studies is shown in Figure 1. The 12-item “Template for Intervention Description and Replication” checklist describes the final pool of SMS text messages [60] (Multimedia Appendix 2).

### Study 1: Behavior Change Expert Workshop

#### Demographics

Of the 10 participants, 8 (80%) were research scientists and 2 (20%) were research scientists and health care professionals. Participants had been in paid research-related posts for between 10 and 25 (mean 16.3, SD 4.8) years. Each participant had published between 3 and 71 papers related to behavior change, medication adherence, and breast cancer (mean 36.1, SD 21.1). Behavior change interventions were the research focus for most participants (8/10, 80%). Half of the participants (5/10, 50%) described medication adherence as central or somewhat central to their work, and 1 participant described breast cancer as central to their work.

#### SMS Text Message Generation

In total, 189 SMS text messages were created for the 6 BCTs during the expert workshop (Table 1).

**Table 1:** Generation and refinement of SMS text messages in study 1.

Behavior change technique	Messages created in workshop, n	Coherence^a^, mean (SD)	Messages removed (research team)^b^, n
Restructuring the physical environment	42	7.5 (1.0)	18
Adding objects to the environment	27	7.4 (1.1)	13
Habit formation	33	8.3 (0.8)	17
Prompts/cues	34	7.8 (0.9)	18
Action planning	28	7.6 (0.7)	15
Self-monitoring of behavior	25	7.9 (0.8)	11

^a^Coherence score ranged from 1 to 10, with higher scores indicating better coherence.

^b^Reasons for message removal include unsuitability for the intervention, repetition, scoring <5.5 on coherence to the behavior.

#### Refinement of SMS Text Messages by the Research Team

In total, 92 messages were removed because they were considered unsuitable (Table 1); for example, they seemed unrealistic, confusing, or exceeded 160 characters. Where multiple messages were similar within a BCT (eg, the suggestion to put your medication by your toothbrush; target BCT: restructuring the physical environment), the messages were combined and agreed upon by the research team (SMCG, SGS, LHH, and ER). On the basis of research team’s ratings of coherence to the behavior, 4 messages were removed: 2 from “restructuring the physical environment,” 1 from “habit formation,” and 1 from “self-monitoring of behavior.”

#### Postworkshop Survey Message Decisions

We removed 3 messages because they scored below the midpoint (5.5) on the 1 to 10 fidelity scale. These are related to “action planning” and “restructuring the physical environment” (Table 2). Messages related to the BCTs “action planning,” “prompts/cues,” and “habit formation” were considered the most relevant when targeting medication adherence. The individual messages rated highest on fidelity were “Buy yourself an attractive pillbox for your medication” (target BCT: “adding objects to the environment”; mean 9.1, SD 1.1) and “At the end of each day, try ticking off whether you have taken your medication in a diary or calendar, to help you keep track” (target BCT: “self-monitoring of behavior”; mean 9.1, SD 0.9). A total of 94 messages remained after study 1.

**Table 2:** Postworkshop survey behavior change expert ratings.

Behavior change technique	Relevance^a^, mean (SD)	Aim to have 12 to 15 messages that reflect the behavior change technique well^b^, mean (SD)	Fidelity^c^, mean (SD)	Fidelity after exclusions, mean (SD)
Action planning	9.0 (0.9)	7.1 (1.5)	6.9 (1.2)	7.3 (0.9)
Prompts/cues	9.2 (0.8)	8.4 (0.7)	8.1 (0.7)	—^d^
Habit formation	9.4 (0.8)	8.1 (1.4)	7.8 (0.8)	—
Restructuring the physical environment	7.9 (1.1)	6.6 (2.1)	6.8 (0.7)	6.9 (0.7)
Self-monitoring of behavior	7.8 (1.4)	8.3 (1.2)	8.1 (0.6)	—
Adding objects to the environment	7.8 (1.5)	7.6 (1.3)	7.7 (1.0)	—

^a^Relevance scores ranged from 1 to 10, with higher scores indicating that the behavior change technique was more relevant to medication adherence.

^b^Scores ranged from 1 to 10, with higher scores indicating that the aim of generating 12 to 15 messages reflecting the behavior change technique had been met.

^c^Fidelity scores ranged from 1 to 10, with higher scores indicating better fidelity to the intended behavior change technique.

^d^No message excluded.

### Study 2: Patient and Public Focus Group

#### Demographics

In total, 5 women aged between 41 and 79 years participated in the focus group (Table 3). All participants were White, had been taking AET for an average of 7 years, and reported using their mobile phone more than once a day.

**Table 3:** Demographics and clinical characteristics of study 2 and 3 participants.

		Study 2 (n=5)	Study 3 (n=60)
Age (years), mean (SD)		54 (15)	51 (16)
Time since diagnosis (years), median (range)		8 (2-20)	2 (0-41)
**Ethnicity, n (%)**			
	White British	5 (100)	49 (82)
	Asian or Asian British	0 (0)	7 (12)
	Black or Black British (African)	0 (0)	2 (3)
	Black or Black British (Caribbean)	0 (0)	1 (2)
	Mixed	0 (0)	1 (2)
**Educational level, n (%)**			
	General Certificate of Secondary Education or equivalent^a^	2 (40)	5 (8)
	National vocational qualification level 1 and 2 (NVQ1+2)	1 (20)	8 (13)
	A-level	0 (0)	6 (10)
	Higher educational qualifications (below degree)	0 (0)	12 (20)
	Degree-level education	2 (40)	25 (42)
	No formal qualifications	0 (0)	3 (5)
	Still studying	0 (0)	1 (2)
Number of children, median (range)		2 (0-2)	2 (1-7)
**Marital status, n (%)**			
	Single	1 (20)	1 (2)
	Married or living together	4 (80)	47 (78)
	Divorced or separated	0 (0)	10 (17)
	Widowed	0 (0)	2 (3)
**Menopausal status, n (%)**			
	Premenopausal	1 (20)	26 (43)
	Postmenopausal	1 (20)	29 (48)
	Unsure	2 (40)	5 (8)
	Other	1 (20)	0 (0)
**Stage of breast cancer at diagnosis, n (%)**			
	1	0 (0)	24 (40)
	2	3 (60)	16 (27)
	3	1 (20)	9 (15)
	4	0 (0)	1 (2)
	Unsure	1 (20)	10 (17)
**Treatment received, n (%)**			
	Surgery: lumpectomy	1 (20)	33 (55)
	Surgery: unilateral mastectomy	4 (80)	15 (25)
	Surgery: double mastectomy	1 (20)	8 (13)
	Chemotherapy	4 (80)	26 (43)
	Radiotherapy	4 (80)	36 (60)
	Other	2 (40)	3 (5)
**Hormone therapy prescribed, n (%)^b^**			
	Tamoxifen	4 (80)	23 (38)
	Letrozole	2 (20)	20 (33)
	Anastrozole	0 (0)	14 (23)
	Exemestane	0 (0)	7 (12)
	Other	1 (20)	0 (0)
	Not prescribed any of these	0 (0)	9 (15)
Time since first prescribed hormone therapy to the nearest year, median (range)		7 (1-20)	2 (0-21)
**Frequency of mobile phone use, n (%)**			
	More than once a day	5 (100)	45 (75)
	Once a day	0 (0)	8 (13)
	More than once a week but not everyday	0 (0)	3 (5)
	Once a week	0 (0)	0 (0)
	More than once a month but not weekly	0 (0)	2 (3)
	Less than once a month	0 (0)	2 (3)
**Frequency of SMS text message use, n (%)**			
	More than once a day	2 (40)	31 (52)
	Once a day	0 (0)	9 (15)
	More than once a week but not everyday	3 (60)	11 (18)
	Once a week	0 (0)	2 (3)
	More than once a month but not weekly	0 (0)	0 (0)
	Less than once a month	0 (0)	7 (12)

^a^Includes General Certificate of Education Ordinary Level (O level) and Certificate of Secondary Education (CSE).

^b^Total is >100% because some participants switched medications.

#### Decisions for Message Development

All suggestions from the focus group in which there was no disagreement between participants were implemented, resulting in the removal of 13 messages (Multimedia Appendix 3). Amendments were made to the wording of certain messages. Example suggestions included using less directive wording, which resulted in phrases such as “you could...” being added. All references to AET were standardized to use “medication,” as agreed by focus group members and researchers.

### Study 3: Patient Survey

#### Demographics

In total, 60 women with ER+ breast cancer completed the web-based survey (Table 3). The average time that the participants had been prescribed hormone therapy was 2 years. Most of the women (53/60, 88%) used their mobile phones at least once per day.

#### Decisions for Message Development

All individual messages and BCTs scored above the midpoint on the acceptability scale (3); therefore, none were removed (Table 4). The mean acceptability ratings for individual messages ranged from 3.52 to 4.28 (scale 1-5). The message scoring highest on acceptability was “Try keeping your medication somewhere visible so that you are reminded to take the medication every day,” targeting the BCT “prompts/cues” (mean 4.28, SD 0.99). The message rated lowest on acceptability was “If you find it hard to remember whether you’ve taken your medication, buying an electronic medication dispenser could help,” targeting the BCT “adding objects to the environment” (mean 3.52, SD 1.11).

**Table 4:** Study 3 acceptability ratings and study 4 fidelity ratings per behavior change technique.

Behavior change technique	Study 3	Study 4		
	Acceptability^a^, mean (SD)	Fidelity^b^, mean (SD)	Messages removed, n	Fidelity after exclusions^b^, mean (SD)
Action planning	3.9 (0.9)	6.9 (1.7)	4	8.0 (1.3)
Prompts/cues	3.9 (0.9)	8.2 (1.2)	1	8.4 (1.3)
Habit formation	4.0 (0.9)	7.0 (1.3)	3	7.8 (1.2)
Restructuring the physical environment	3.9 (0.8)	6.9 (1.2)	3	7.2 (2.2)
Self-monitoring of behavior	3.9 (0.9)	8.0 (1.3)	0	—^c^
Adding objects to the environment	3.8 (0.9)	7.3 (1.7)	2	7.8 (1.8)

^a^Acceptability score ranged from 1 to 5, with higher scores indicating better acceptability.

^b^Fidelity scores ranged from 1 to 10, with higher scores indicating better fidelity to the intended behavior change technique.

^c^No messages were removed.

### Study 4: Expert Survey

#### Demographics

In total, 12 experts in behavior change participated in the survey: 11 were research scientists and 1 was a research scientist and health care professional. All participants described behavior change interventions as central or somewhat central to their work. A total of 5 participants described medication adherence as central or somewhat central to their work. In addition, 3 participants described breast cancer as central or somewhat central to their work. The participants had been in paid research-related posts for between 5 and 16 (mean 9.3, SD 3.3) years and had published between 5 and 25 papers related to medication adherence, behavior change, and breast cancer (median 10.5).

#### Decisions for Message Development

We removed 13 messages because they scored below the midpoint (5.5) of fidelity to the intended BCT (scale 1-10; Table 4). The 2 highest-scoring messages were “If you notice your medication is low, you could leave the empty box on the kitchen table to remind you to call the pharmacy,” targeting the BCT “prompts/cues” (mean 9.25, SD 0.87), and “As a suggestion, when you brush your teeth in the morning, follow it immediately by taking your medication,” targeting the BCT “action planning” (mean 9.25, SD 0.75). After removing messages scoring below the fidelity threshold, the 2 messages with the lowest fidelity were “If you take your medication in different places you may be more likely to forget to take it. By leaving your tablets in one place you’re less likely to forget,” targeting the BCT “restructuring the physical environment” (mean 5.58, SD 3.26), and “If you might forget to collect your prescription, you could add an appointment into your phone calendar to remind you,” targeting the BCT “adding objects to the environment” (mean 5.58, SD 2.81).

### Clinical Review

We removed 2 messages and amended 3 messages following the advice of a consultant pharmacist (Table 5). Messages related to taking the AET tablet with hot drinks were removed, as this is not recommended.

**Table 5:** Reasons for message amendments or removal following clinical review.

Behavior change technique	Message	Action	Reason	Amended message
Action planning	“If you take your medication with a hot drink in the morning, then try getting the medication when you are boiling the kettle.”	Removed	Medication is recommended to be taken with a glass of water, not hot drinks	—^a^
Habit formation	“Taking your medication can be as routine as a morning coffee. Try taking your medication at the same time in your routine so it becomes easier to remember.”	Removed	Not recommended to take medication with hot drinks—message could imply this.	—
Restructuring the physical environment	“As a suggestion, always keep some spare medication in your bag/coat, just in case you realise you haven’t taken them later on that day and are not at home.”	Amended	Not recommended to put medication in the coat pocket, as it is easy to fall out.	“As a suggestion, always keep some spare medication in your bag, just in case you realise you haven’t taken them later on that day and are not at home.”
Prompts/cues	“If you notice your medication is low, you could leave the empty box on the kitchen table to remind you to call the pharmacy.”	Amended	You need to ring the GP^b^ practice for a repeat prescription, not the pharmacy.	“If you notice your medication is low, you could leave the empty box on the kitchen table to remind you to call the GP.”
Prompts/cues	“Notice your medication is nearly out in the evening? We suggest that you put a Post-it note on the bathroom mirror to call the pharmacy in the morning.”	Amended	You need to ring the GP practice for a repeat prescription, not the pharmacy.	“Notice your medication is nearly out in the evening? We suggest that you put a Post-it note on the bathroom mirror to call the GP in the morning.”

^a^Message was removed, not amended.

^b^GP: general practitioner.

### Final Pool of Messages

After all the studies, a pool of 66 messages remained (examples in Multimedia Appendix 4). The full pool of messages is available to research teams upon reasonable request. All messages are 1-way and are designed to be sent by an automated message system. Overall, the messages have a readability score of 8.2 on the Flesch-Kincaid reading grade level scale, which corresponds to the reading age of an eighth grader (aged 13 years) [61].

## Discussion

### Principal Findings

In 4 linked studies involving behavior change experts and women with breast cancer, we developed a pool of 66 SMS text messages to promote habitual medication taking. The text messages were considered acceptable to women with breast cancer and had adequate fidelity to the target BCTs. The BCTs were chosen based on habit theory to promote context-dependent repetition of medication-taking behaviors so that habits may form. Next, we will examine the extent to which these messages can support adherence to AET in women affected by breast cancer [55]. If SMS text messages are effective in improving AET, they could form part of a multicomponent support program for women with breast cancer.

This study builds on previous attempts to use SMS text messaging interventions in women taking AET by developing explicitly theory-based messages. Previous SMS text messaging interventions aimed at improving AET adherence have produced equivocal findings [45-47]. One trial reported statistically significant effects at 6-month follow-up (*P*=.03), but these effects were not sustained at 12 months (*P*=.62) [46]. This null result at longer follow-up may be explained by the use of atheoretical, simple text prompts. Although simple prompts may be effective for short-term behavior change, they may create reliance on the text message as a prompt. In turn, behavior change may not be sustained upon message cessation. To address this issue, our messages encourage taking medication in the same context repeatedly to form cue-behavior associations, in line with habit theory [28-33]. Forming context-dependent associations for medication taking can lead to sustained behavior change even if the messages are stopped [28-33].

Our iterative text message development process using 4 interlinked studies enabled us to continually optimize the pool of messages throughout the development process. We used the key steps recommended by the intervention development guidelines [51]. For example, we included stakeholder involvement at multiple points throughout the development process, drew on existing theories, and continually refined the intervention. This advances on previous interventions that tend to be limited in their reported development process and therefore lack justification for the content of the intervention. Owing to our process, our pool of text messages quantitatively demonstrated acceptability in our target population and adequate fidelity to the intended BCTs. Consequently, the messages could be used to test habit theory in future evaluations.

The final pool of text messages demonstrated prospective acceptability in women with breast cancer. This was based on a single assessment of acceptability. Our approach was effective in ensuring that no messages considered unacceptable to women with breast cancer were included in the final pool of text messages. Previous studies involving SMS text messages to support adherence to diabetes medications found prospective and experienced acceptability to be correlated [54]. In the subsequent evaluation of these messages, our focus will turn to assessing experienced acceptability, including the satisfaction and usefulness of the messages when delivered over a prolonged period [54]. The theoretical framework of acceptability conceptualizes acceptability as a multifaceted construct composed of 7 components and could therefore provide a useful basis for the assessment of experienced acceptability [62].

Despite our rigorous intervention development process, some key uncertainties remain. First, there are uncertainties surrounding how these text messages should be used in an intervention; which messages to use, the frequency with which messages should be sent, and the duration for which they need to be sent to support habit formation [63]. Such uncertainties surrounding implementation could be explored further to build an optimal intervention using our pool of SMS text messages. Our approach to implementation, including justifications for our proposed frequency of messages, is explained elsewhere [26]. Second, some messages may show fidelity to other BCTs in the BCTTv1 outside of the 6 specified BCTs they were generated to target, for example, “problem-solving.” To aid transparency, the messages chosen to be used in any intervention should be coded using the BCTTv1 to reflect any additional BCTs that the messages may target [26,48].

### Strengths and Limitations

We adapted an established approach previously used to develop SMS text messages to support diabetes self-management and demonstrated how the process can be conducted remotely [53]. Our approach can act as a guide for other researchers conducting remote codevelopment work. However, our study had limitations. In the web-based workshop in study 1, it was difficult to facilitate conversations between behavior change experts to collaboratively generate SMS text messages as planned. Consequently, the messages were largely developed individually. To encourage more collaboration during the workshop, researchers could consider allocating a period devoted to anonymously editing other participants’ messages on the web-based platform (eg, Padlet), which may facilitate collaboration better than discussion alone. In addition, all women (5/5, 100%) in study 2 and most women (49/60, 82%) in study 3 were of White ethnicity. This limits generalizability, as the acceptability of digital health interventions may be influenced by ethnicity and cultural norms [64]. Further developmental studies are needed to explore the acceptability and appropriateness of these messages in a wider range of sociodemographic groups.

### Conclusions

In conclusion, we conducted a series of 4 linked studies using mixed methods to develop a pool of 66 brief messages to support medication adherence to AET in women with breast cancer. The messages were based on 6 BCTs theorized to support habit formation. The messages were rated as acceptable to women with breast cancer and showed fidelity to the intended BCTs. Further evaluation of these messages is needed to establish whether they can support medication adherence behaviors.

## Appendix

Multimedia Appendix 1 Study 2 focus group schedule.

Multimedia Appendix 2 Template for Intervention Description and Replication checklist.

Multimedia Appendix 3 Justifications for deleting messages following study 2.

Multimedia Appendix 4 Example SMS text messages from the final pool.

## Abbreviations

AET: adjuvant endocrine therapy
BCT: behavior change technique
BCTTv1: version 1 of the behavior change taxonomy
ER+: estrogen receptor–positive

## Data Availability

The data generated in this series of studies are not publicly available because they contain the full list of SMS text messages, which have not been publicly shared to protect our intellectual property. The data and the final list of SMS text messages are available from the corresponding author on reasonable request.
