# Supplementary material for: Codevelopment of a Text Messaging Intervention to Support Adherence to Adjuvant Endocrine Therapy in Women With Breast Cancer: Mixed Methods Approach
Source: J Med Internet Res. 2023 May 24;25:e38073. doi: 10.2196/38073 (PMC10248768; doi:10.2196/38073)
Supplement: Multimedia Appendix 1 [file jmir_v25i1e38073_app1.docx]

**Supplementary File 1: Study 2 Focus Group Schedule**

**Introduction**

- The consent form will be read out and any participants who disagree with the statements will be able to leave the call.
- Aims of the focus group; to discuss the wording of SMS text messages generated by research scientists with the aim to support medication adherence in breast cancer patients
- Structure of the session; introduce a behaviour change technique, read out SMS messages relating to that behaviour change technique, and then to discuss wording of these messages.
- Right to withdraw at any time

**Discussion of SMS Messages**

Each Behaviour change technique will be introduced, giving the name and a short description of BCT, based on the v1 taxonomy. For example:

Name: Action Planning

Description: These messages have been created in order to prompt detailed planning of performance of the behaviour (including at least one of context, frequency, duration and intensity). Context may be environmental (physical or social) or internal (physical, emotional or cognitive)*.* This also includes “implementation intentions” which are If….then… plans.

The SMS messages relating to this BCT will be read out and displayed on the screen, and there will be a discussion regarding the SMS messages. Discussion points will include:

- What do you think about the wording of these SMS messages?
- Is there anything you would change about the wording in any of these messages?
- Are there any messages that you would not want to receive and why?

This process will be repeated for each BCT.

**Conclusion**

Participants will be thanked for their time and informed that they will be sent a debrief letter.
