# Supplementary material for: Codevelopment of a Text Messaging Intervention to Support Adherence to Adjuvant Endocrine Therapy in Women With Breast Cancer: Mixed Methods Approach
Source: J Med Internet Res. 2023 May 24;25:e38073. doi: 10.2196/38073 (PMC10248768; doi:10.2196/38073)
Supplement: Multimedia Appendix 2 [file jmir_v25i1e38073_app2.docx]

## Supplementary File 2- The TIDieR (Template for Intervention Description and Replication) Checklist:

| **N^o^** | **What** | **Details** |
| --- | --- | --- |
| **1** | **Name** | Brief SMS messages to support medication adherence to adjuvant endocrine therapy in women with breast cancer |
| **2** | **Why: Rationale, theory, goal** | Adjuvant endocrine therapy (AET) is routinely prescribed to women with early stage breast cancer for 5-10 years once active hospital-based treatment has ended. AET prevents breast cancer recurrence and mortality. However, up to three-quarters of women prescribed AET are non-adherent. Unintentional nonadherence (e.g. forgetting to take your medication) is common in women taking AET. Previous interventions to support AET adherence tend to consist solely of educational based interventions, are not grounded in theory, and lack transparency in their development process.  Promoting habits surrounding medication taking could improve unintentional nonadherence as medication taking will be less reliant on memory alone. SMS based interventions are a potential method to improve adherence and have been shown to be effective in other chronic illnesses. Therefore, we developed a pool of SMS messages to support adherence to AET that are based on habit formation theory. |
| **3** | **What Materials** | We developed a pool of 66 messages. Examples of these are available in Multimedia Appendix 4. The full pool of messages is available to research teams upon reasonable request. The pool contains the following messages based on selected BCTs from the BCTTv1. All messages are below 160 characters.   - 17 messages targeting ‘Restructuring the physical environment’ - 10 messages targeting ‘Adding objects to the environment’ - 9 messages targeting ‘Habit formation’ - 13 messages targeting ‘Prompts and cues’ - 6 messages targeting ‘Action Planning’ - 11 messages targeting ‘Self-monitoring of behaviour’   The messages chosen to be used, and the frequency and duration of messages to be sent can be determined by intervention developers. |
| **4** | **What Procedures** | The SMS messages are designed to be delivered to a participant’s mobile phone device. |
| **5** | **Who provided** | The SMS messages are designed to be delivered by an automated system. |
| **6** | **How: mechanisms of delivery** | The SMS messages are designed to be sent in an automated fashion to a participant’s mobile device. |
| **7** | **Where: location of delivery** | The SMS messages are designed to be delivered to a mobile device. All messages are under 160 characters to enable this. |
| **8** | **When and how much** | N/A. The current study has generated a pool of SMS messages that could be used in interventions with varying time periods. |
| **9** | **Tailoring** | N/A |
| **10** | **Modifications** | The pool of messages was modified following each individual study. Following study 1, messages scoring below 5.5 on the fidelity subscale were removed. Following study 2, messages deemed unacceptable were removed. Following study 3, no messages were removed as no messages scored below 3 on the acceptability rating. Following study 4, messages scoring below 5.5. on the fidelity subscale were removed. |
| **11** | **How well (planned)** | Messages were assessed for fidelity to the intended BCTs by experts in behaviour change in studies 1 and 4. Experts in behaviour change were asked to rate each SMS message based on how well it targeted the BCT it was intended to target. Messages were rated on a scale of 1(not very well) to 10 (very well). Any messages scoring a mean of below 5.5 (the midpoint on the fidelity scale) were deemed to have low fidelity and were removed from the pool of messages. |
| **12** | **How well (actual)** | N/A |
