# Supplementary material for: Codevelopment of a Text Messaging Intervention to Support Adherence to Adjuvant Endocrine Therapy in Women With Breast Cancer: Mixed Methods Approach
Source: J Med Internet Res. 2023 May 24;25:e38073. doi: 10.2196/38073 (PMC10248768; doi:10.2196/38073)
Supplement: Multimedia Appendix 3 [file jmir_v25i1e38073_app3.docx]

Supplementary File 3- Justifications for deleting messages following Study 2.

| Behaviour Change Technique | Message | Quote from focus group |
| --- | --- | --- |
| Restructuring the physical environment | On days when you need to pick up your medication, place an empty medication pack in your purse as a reminder to pick your medication | “I don’t know how big people’s purses are, but you know, by all means put it in your handbag or put it on a shelf by the back door, or but yeah, I just wasn’t sure about saying to put it in your purse.” |
|  | Store your medication on top of your usual breakfast items so that you remember to take them with breakfast | “some meds you’re not meant to take with food” |
|  | If leaving the house overnight then make life easier for yourself by putting your tablets somewhere you will see them to help remind you to take them | “that’s a one-off, it’s not like, oh you’re going to be leaving the house every night, so I need to form a habit to leave the house every night overnight, do you know what I mean, it doesn’t seem to fit there.” |
| Adding objects to the environment | What object could you put in your house to remind you to take your medication? | “I didn’t really sort of see the point, in a way it says an object, what are you going to get that’s going to remind you other than leaving your pill box out, where you can see it. I just couldn’t think of what you would use as an object to remind yourself” |
|  | Buy your favourite drink to wash down your medication | “you know, lots of people would just wash it down with water, it’s a small pill isn’t it. I suppose it would work, there’s some people maybe don’t like water, so maybe buying your favourite drink would…a little bit pointless, so that’s just me personally” |
| Habit formation | Try to always take your meds with your first meal of the day, so that it becomes part of your everyday routine | “try to take your meds with the first food of the day, some meds you’re not meant to take with food.” |
|  | A little poem, for you to commit to today, I'll take my medication during, the same activity every day | “I personally would just find that a little bit patronising, …., I think there’s enough other messages which are kind of stronger than that, and if you have to kind of whittle it down to the, to certain ones I wasn’t a fan of that one.” |
|  | Form a habit of putting an old empty meds packet in your purse, as reminder to collect your next prescription | “And then form a habit of putting an empty meds packet in your purse, I don’t know how big people’s purses are, but you know, by all means put it in your handbag or put it on a shelf by the back door, or but yeah, I just wasn’t sure about saying to put it in your purse.” |
| Prompts and Cues | Keep your medication next to coffee/tea in kitchen cupboard to remind you to take it with your first cuppa. | “I think you have to be careful of that, because my, one lot of things I, something I have to take you can’t take it with tea or coffee.”  New speaker: “I think all the store medications it says keep out of the reach of children, if you’re sticking something on a worktop that’s potentially within reach of a child, just need to be careful about giving directions like that I thought.” |
|  | Before taking the glass of water you may keep by your bed downstairs - stop and check if you have taken your medication that day | “it’s kind of like that presumption that everyone goes upstairs with a glass of water, if you’re taking, you know, you have got a glass of water so before you take it downstairs check that you’ve had your medication” |
| Self-monitoring of behaviour | At the end of each day, try ticking off whether you have taken your medication in a diary or calendar, to help you keep track | “I take mine on a morning, so by the time the end of the day comes with having chemo fog brain I couldn’t remember whether I’d taken it or not. Well for me that was just a little bit is it worth putting in, you know, there are quite a few ladies that do suffer memory loss after they’ve had treatment, so I don’t know, if you’re doing something in the morning, trying to remember whether you’ve done it later on is just a bit…Yeah, not for me, but I mean some [laughs], with some people it might be alright for, but it did stand out that that one was a bit…” |
|  | One way to help reduce forgetting to take your medication is to simply record each day if you took it or not. | “it’s quite, 1) what’s the point, remember your tablet, don’t remember to write down you’ve forgotten it, so to me it's like kind of warped logic. But also it’s quite negative, I think it should kind of be focusing on you are going to do this, forgetting is not an option, but don’t… let’s kind of go from a positive 100% point and sort of slide from that, not that oh you obviously will forget a few days, make sure you write it down and see if there’s a link or whatever, so I just had a problem with that whole one to be honest” |
|  | To monitor your meds you could try writing the first letter of each day next to each pill on your blister pack (M=Monday). This will track how you're doing | “I think number five is probably unachievable, so probably not worth the text message, I don’t think there’s space on your foil to get a, you know, to get a Sharpie and write the day of the week, and if anything that would be a good one to go back to the manufacturers of the medication, if they see it so often, that yeah it would be great if it could have days on the week just like the contraceptive pill does, but yeah, I’m not sure, I kind of think that would probably be a wasted text message because I can’t imagine anybody would try and write the days of the week on the foil packet.” |
