# Supplementary material for: Codevelopment of a Text Messaging Intervention to Support Adherence to Adjuvant Endocrine Therapy in Women With Breast Cancer: Mixed Methods Approach
Source: J Med Internet Res. 2023 May 24;25:e38073. doi: 10.2196/38073 (PMC10248768; doi:10.2196/38073)
Supplement: Multimedia Appendix 4 [file jmir_v25i1e38073_app4.docx]

**Supplementary File 4- Example SMS Messages**

Behaviour change techniques (BCTs) are based on the behaviour change taxonomy (v1).

| **BCT** | **Example Message** |
| --- | --- |
| **Restructuring the physical environment** | Try putting your medication by something you do everyday e.g. your toothbrush. As long as you clean your teeth every day, you won't forget to take them! |
| **Adding objects to the environment** | Why not try buying yourself an attractive pillbox for your medication, to help you to remember to take it. |
| **Habit formation** | We suggest you always order your prescription in the same way (e.g. using an app) and in the same place (e.g. in your living room) so that it becomes a habit. |
| **Prompts and cues** | Try keeping your medication somewhere visible so that you are reminded to take the medication every day. |
| **Action Planning** | As a suggestion, when you brush your teeth in the morning, follow it immediately by taking your medication. |
| **Self-monitoring of behaviour** | Making a note when you've taken your medication can help keep you on track. You could make a note in a calendar or diary, or use electronic notes or an app. |
